# UPDATED SYSTEMATIC REVIEW TO IDENTIFY INTERVENTIONS TO COMBAT ELDER NEGLECT TARGETING CAREGIVERS

**DOI:** 10.1093/geroni/igae098.2056

**Published:** 2024-12-31

**Authors:** Sophie Park, Helena Costantini, Amy Shaw, Alyssa Elman, Tony Rosen, Sara Czaja

**Affiliations:** Weill Cornell Medicine/Center on Aging and Behavioral Research, New York, New York, United States; Weill Cornell Medicine/Center on Aging and Behavioral Research, New York, New York, United States; Weill Cornell Medicine, New York, New York, United States; Weill Cornell Medicine, New York, New York, United States; Weill Cornell Medicine, New York, New York, United States; Weill Cornell Medicine/Center on Aging and Behavioral Research, New York, New York, United States

## Abstract

Elder neglect is a subtype of elder mistreatment that is associated with serious medical and social consequences including mortality. Elder neglect by a caregiver is common among older adults receiving assistance from informal caregivers. Few interventions that target informal caregivers have focused on mitigating elder neglect. We updated an existing 2018 systematic review of elder mistreatment interventions, focusing specifically on interventions addressing elder neglect and targeting caregivers. The goal was to examine methods of intervention strategies to guide the development of a new caregiver intervention. The existing and updated review followed PRISMA guidelines and included 6 databases. Across both reviews, only 20 programs focused on elder neglect and targeted informal caregivers. Most programs used psychoeducation/therapy/counseling (65%) and/or education (30%), and 25% were multi-component interventions. Only one program was found to be very likely and two programs likely to be implementable in a low-resource environment. A formal program evaluation was included in 70% of the programs; only 15% were rated as having a high-quality study design. These findings suggest that few programs have been developed to address the important issue of caregiver elder neglect. The design of future interventions should focus on programs that are feasible to implement in low-resource environments and that contain rigorous evaluation strategies. The study findings also suggest that important areas of intervention focus include enhancing caregiver preparedness and reducing caregiver burden. Overall, there is a clear need for caregiver elder neglect interventions, especially those that can be implemented in low resource environments.

